# Pharmacotherapy for obesity: moving towards efficacy improvement

**DOI:** 10.1186/s13098-023-01233-4

**Published:** 2024-01-03

**Authors:** Walmir Coutinho, Bruno Halpern

**Affiliations:** 1 State Institute of Diabetes and Endocrinology, Rua Moncorvo Filho, 90, Rio de Janeiro, RJ 20211-340 Brazil; 2 Department of Medicine, Pontifical Catholic University of Rio de Janeiro, Rua Marquês de São Vicente, 225, Gávea, Rio de Janeiro, RJ 22541-041 Brazil; 3 Department of Endocrinology, Obesity Unit, Hospital das Clínicas Faculdade de Medicina da Universidade de São Paulo. Av. Dr. Enéas de Carvalho Aguiar, 255, 7Th Floor, Room 7037, São Paulo, SP 05403-000 Brazil

**Keywords:** Obesity, Weight loss, Anti-obesity drugs, Sibutramine, Orlistat, GLP-1 analogs, Liraglutide, Semaglutide

## Abstract

Obesity is a chronic, recurring, progressive disease and a major public health problem associated with several other diseases that lead to disability, morbidity, and mortality. The prevalence of obesity has increased at pandemic levels, along with increasing weight-related comorbidities and deaths worldwide. Lifestyle interventions alone provide clinically significant long-term weight loss in only a small proportion of individuals, and bariatric surgery is not suitable or desirable for all patients. Historically, anti-obesity medications achieved a mean efficacy with weight loss between 5 and 10%, which significantly impacted several comorbidities and risk factors, but the average efficacy of these medications remained lower than that expected by both patients and health care professionals and eventually curbed long-term use. Moreover, there is no direct evidence on the impact of anti-obesity medications on cardiovascular outcomes. Semaglutide is a newer anti-obesity medication that changes the overall landscape, as phase 3 studies show a mean weight loss near the 15% threshold and significant proportions of patients with a weight loss of greater than 20%. In this review, we focus on the currently available anti-obesity medications, discuss the results of semaglutide, and present perspectives on the future of obesity treatment after semaglutide.

## Background

Obesity is a public health problem in developed and developing countries and is predicted to affect over a billion people worldwide by 2030 [1]. Individuals with obesity have increased risk of other morbidities, including a higher risk for at least 13 types of cancer [2], higher risk of all-cause mortality, disability, and loss of productivity [3–5].

Obesity also has a significant economic impact through direct and indirect health care costs. A multicountry study on the economic impacts of obesity showed a significant hindrance in economic development, as the estimated obesity costs were between 0.80% and 2.42% of gross domestic product (GDP) in 2019 [6]. If this trend continues without significant change until 2060, the economic impact of obesity is estimated to reach 3.6% GDP on average [6]. In Brazil, the estimated public and private health care expenditure is US$ 14 billion for 26 obesity-related diseases [6].

Obesity is a chronic and recurring disease. Unfortunately, this concept is not widely accepted, thus reducing access to care. The ACTION-IO study, including 14,502 people with obesity and 2,785 health care professionals, showed that although most people with obesity recognized it as a disease, they usually did not seek medical care [7]. Only half of the patients with obesity discussed their weight with a health care professional in the last five years; only one-third received a diagnosis of obesity, and only one in five had a follow-up appointment to evaluate treatment progress [7]. It took almost six years from the first weight loss attempt to the first conversation about weight with a health care professional [7]. Health care professionals do not discuss weight with their patients because they think that the patients are not motivated; nonetheless, more than 80% of patients have made at least one serious attempt to lose weight in the last 3 years [7].

Even modest weight loss, such as 2 to 5%, is associated with health and quality of life benefits; however, several guidelines recommend a 5–10% weight loss [8–11]. As illustrated in Table 1, the percentage of weight loss is directly related to improved comorbidities [12]. Although direct evidence of reduced cardiovascular outcomes from clinical data is not yet available, a post hoc analysis of the LOOK AHEAD trial suggested that a weight loss of at least 10% is associated with a 21% reduction in cardiovascular events [13]. Weight loss over 10% reduces intra-abdominal adipose tissue and inflammation [14], and indirect data from bariatric surgery cohorts, such as the SOS study, suggest that 16% weight loss reduces mortality [12, 14].

**Table 1:** The effect of weight loss on obesity-related comorbidities

Percentage of weight loss	Effect on comorbidities
≥ 2.5%	Prevention of diabetes in impaired blood glucose Improved T2DM
	Decreased plasma levels of triglycerides
	Improved ovulation and pregnancy in patients with polycystic ovarian syndrome and infertility
From 5% to < 10**%**	Increased HDL
	Improved function and pain of patients with knee osteoarthritis
	Improved symptoms of urinary incontinence
	Decreased risk of depression
	Improved sexual dysfunction
	Improved quality of life
	Decreased costs of hospitalization and medications
≥ 10%	Improved sleep apnea
	Decreased risk of cardiovascular events
	Improved NASH
≥ 16%	Reduced risk of mortality

Adapted from Ryan et al., 2017 [12]. T2DM: type-2 diabetes mellitus. *HDL* high-density lipoprotein, *NASH* nonalcoholic steatohepatitis

Nonetheless, weight loss is not as simple as has been claimed. According to an extensive population analysis, only 1 in 210 males and 1 in 124 females with a BMI between 30 and 35 kg/m^2^ achieve normal weight without bariatric surgery; for people with a BMI > 40 kg/m^2^, the numbers are worse: only 1 in 1220 males and 1 in 677 females achieve normal weight [15]. Lifestyle changes alone result in a mean body-weight loss of less than 5% of body weight in the long term. A meta-analysis of 14 different intervention trials assessing the use of diet and exercise for weight management showed that most patients regained most weight lost after a follow-up from 4 to 7 years [16].

Currently, the most effective treatment for obesity is bariatric surgery; in prospective studies, individuals who underwent bariatric surgery had reduced mortality, myocardial infarction, and T2DM incidence [14, 17, 18]. However, bariatric surgery is restricted to class II or III obesity, and even in this population, it is not suitable or acceptable for all patients due to fears of long-term complications, stigma, and difficulties with access [19].

**Fig. 1:**
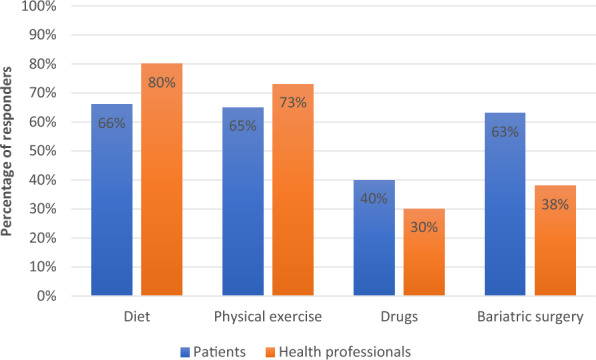
Opinions of patients and health professionals regarding the most effective treatment for obesity. Data from Caterson et al., 2019 [7]

Treatment with anti-obesity medications (AOMs) is generally associated with greater weight loss than lifestyle treatment alone, and a prerequisite for approval of any AOM is also its effectiveness in long-term weight maintenance. AOMs remain underused, as the stigmatization of these drugs discourages their prescription. Older drugs that had limited efficacy and uncertain long-term safety led to concerns that are still present in the medical community [20, 21]. The likelihood of a clinician prescribing a medication for T2DM is 15 times greater than that for obesity [22]. In the ACTION-IO study, only 40% of people with obesity and 30% of health care professionals declared their belief in the efficacy of AOMs [7] (see Fig. 1), despite all the accumulated evidence that demonstrated their efficacy. A recent market analysis suggested that nearly 50% of prescribed AOMs are not even initiated [23].

Long-lasting treatments significantly impact complications, and medication withdrawal generally leads to weight regain, which reduces the clinical benefits of treatment. Weight regain after substantial weight loss is a biological process. When weight loss occurs, persistent hunger and appetite increase, in line with the “hypothalamic set-point theory” [24–27]. Part of this effect is related to a disproportionate decrease in leptin levels, a hormone secreted by adipose tissue that, when reduced, leads to increased appetite and reduced thermogenesis [24–26, 28]. Changes in adipose tissue biology also contribute to weight regain because adipocytes reduce their size, but not their number, after weight loss [26, 27]. One seminal study found that in patients with obesity, a 14% weight loss results in changes in the behavior of at least nine hormones, leading to an imbalance favoring an increase in food consumption [29]. This effect lasts for at least one year after diet restriction. These findings are in line with other studies [24–26, 30]. Neural regulatory signaling is also imbalanced after reducing dietary restriction, prevailing in reward-related signaling that induces high caloric food intake [26, 31]. On the other side of the energy balance equation, the energy expenditure related to basal metabolism is decreased by 15–30 kcal/day for each kg of weight lost [32, 33].

The amount of weight loss that is expected by patients with obesity and healthcare professionals is high and difficult to achieve [7]. Bridging the gap between weight loss goals and actual results by improving the efficacy of treatment is imperative. At the same time, it is important to discuss the fact that even smaller amounts of weight loss could lead to health benefits; otherwise, the discontinuation rate of obesity treatments will continue to increase.

A recent proposal for a new classification of obesity could help clinicians and patients to set more realistic goals, which should be discussed individually [34]. The classification based on weight history introduces the term “controlled obesity” for individuals with weight losses higher than 10% (or 15% if BMI over 40 kg/m^2^) and is based on the maximal weight achieved in life (MWAL) and the weight trajectory. The classification distinguishes patients with “unchanged” (if close to the MWAL, or less than 5%), “reduced” (if 5–10% weight loss is achieved) and “controlled” obesity (> 10%) if BMI is between 30–40. For individuals with higher BMIs, the threshold for reduced BMI is > 10%, and the threshold for controlled BMI is > 15%. It is important to point out, however, that this proposal is not intended to be a guideline but an ancillary tool to set targets, and the percentage of weight loss achieved should be mentioned in the classification as well [35].

## Approved AOMs and their efficacy: what is available to date?

### Peripheral action: orlistat

Orlistat is an inhibitor of gastrointestinal lipases that is approved worldwide for chronic weight management. It decreases lipid absorption and promotes a negative caloric balance by partially blocking dietary triglyceride hydrolysis into absorbable monoglycerides and free fatty acids [36, 37].

RCTs with orlistat show 5–10% weight loss of patients with obesity, being superior to placebo in the doses from 30 to 240 mg [38–40]. In a trial including a one-year evaluation period, 9.1% of patients treated with orlistat had a ≥ 20% reduction in the initial body weight versus 6.1% of patients treated with a placebo [40].

A meta-analysis showed a placebo-subtracted weight loss of 2.3 kg with at least one year of treatment with orlistat [41]. A more recent meta-analysis including 16 RCTs assessing the effects of orlistat compared with those of a placebo showed similar results, with 2.6 kg of placebo-subtracted weight loss after one year of treatment [42]. The OR for achieving a reduction of at least 5% of body weight were 2.70, and 2.42 for losing at least 10% of body weight [42].

The longest double-blind RCT assessing the effects of orlistat was the four-year XENDOS trial and showed the efficacy of orlistat on significant weight loss and prevention of T2DM in patients with obesity (n = 3,304) [43]. Regarding metabolic health beyond weight loss itself, in the XENDOS trial, orlistat was associated with a reduced risk of developing T2DM, as well as a reduction in LDL cholesterol [43]. Orlistat reduced the risk of developing T2DM by 37.3%, as the incidence of T2DM in patients treated with orlistat was 9% versus 6.2% with placebo (p = 0.0032) [43]. Orlistat led to a greater reduction in HbA1c (-0.74%) compared with that of placebo (-0.31%; p < 0.0001), which appeared to be independent of the amount of weight lost, as patients with minimal weight loss (≤ 1%) still showed a benefit both in levels of fasting plasma glucose (-0.83 mmol/l) and in HbA1C (-0.29%) [43].

A pooled analysis from two RCTs (n = 909) showed that the relative risk of failure to maintain at least a 10% weight loss after two years of treatment was 0.88 (95% CI 0.83, 0.93) for participants using 120 mg of orlistat three times a day versus those using a placebo (p = 0.00002), and it was 0.90 (95% CI 0.85, 0.95) for participants using 60 mg versus those using a placebo (p = 0.0002) [44].

Concerning safety, orlistat is generally considered safe, with most of the concerns regarding its safety being related to tolerability [45]. Mild-to-moderate gastrointestinal events were the most frequent adverse events associated with orlistat treatment, occurring in up to 91% of patients in the first year of treatment and 36% after four years of treatment [43]. Fecal incontinence, flatulence, and steatorrhea usually improved with a low-fat diet. No data are available on the long-term cardiovascular safety of orlistat, and no trial was designed to evaluate those variables. However, no deaths were related to the use of orlistat during the four-year study [43]. Nephrolithiasis and liposoluble vitamin malabsorption may occur rarely; hepatotoxicity has been described, but doubts remain on whether there is a causal relationship [43, 45].

## Central action by activity on neurotransmitters: monotherapy and combined drugs

### Monotherapy: sibutramine and phentermine

#### Sibutramine

Sibutramine is a central-acting AOM, a potent serotonin and noradrenaline reuptake inhibitor that enhances satiety [46]. It was withdrawn from the market in most countries due to increased nonfatal cardiovascular events demonstrated in the SCOUT study [47]. It remains available for long-term use in some countries, such as Brazil and Russia.

The mean efficacy of sibutramine reported in randomized controlled trials (RCTs) is a 5–10% body-weight loss. RCTs with sibutramine 15 mg showed superiority (greater weight loss) over 10 mg and placebo [48, 49], including patients with obesity and comorbidities [50]. In a double-blind RCT (n = 362), patients with obesity lost 8.3% of body weight after 54 weeks of treatment with 15 mg sibutramine compared with 4.9% with placebo, p < 0.01 [48]. However, there was no available information on ≥ 20% weight loss in any of the cited RCTs. None of the trials evaluated the maintenance or loss of lean and fat body mass.

Sibutramine maintains its efficacy on a long-term basis and prevents weight regain. A total of 43% of patients maintained weight loss with the continuous use of sibutramine 10 to 20 mg for two years versus 16% with placebo [odds ratio (OR) 4.64, p < 0.001] [51]. In a crossover clinical study, 85% of patients switching from sibutramine to placebo experienced a 43% weight regain in a 6-month follow-up [52]. In a RCT including patients with obesity who experienced weight loss after four weeks of a low-calorie diet, the use of 15 mg sibutramine for one year was associated with a mean weight loss of 5.2 kg, while patients in the placebo group regained 0.5 kg (p = 0.004) [53]. A total of 75% of patients with obesity treated with sibutramine maintained weight loss after one year versus 12% of the placebo group (p < 0.001).

The results from a meta-analysis (10 studies; n = 2,623) showed that patients treated with sibutramine for at least one year lost 4.3% more body weight than those treated with placebo, with a placebo-subtracted weight loss of 4.9 kg [41]. Another meta-analysis showed that the mean difference in weight loss between 15 mg sibutramine and placebo was −6.35 kg after 12 months of treatment [54].

Safety concerns lead to the withdrawal of sibutramine in most countries. In the SCOUT study (n = 10,744), patients with obesity and cardiovascular risk had a 16% increase in the relative risk of cardiovascular events in the sibutramine arm compared with the placebo arm (11.4% versus 10%; HR 1.16; 95% CI 1.03,1.31; p = 0.02) [55]. Rates of all-cause mortality were similar between groups. However, a post hoc analysis showed that for each kg of weight lost in either arm of the SCOUT study, there was a 0.8% decrease in the absolute risk of cardiovascular outcomes [55]. In a real-world study, individuals using sibutramine (n = 23,927) experienced an increased risk of a cardiovascular event compared with those using orlistat (n = 77,047) in patients with cardiovascular disease (HR 4.37; 95% CI 2.21,8.64); however, there was no difference in all-cause mortality, and patients without preexisting cardiovascular disease did not show a higher risk for a cardiovascular event [56].

#### Phentermine

Phentermine is an atypical amphetamione analogue, acting in central nervous system by inhibiting appetite through norepinephrine agonism. It is approved for obesity treatment in the United States and several other countries for short-term use (only 12 weeks), due to a lack of long-term studies. However, real world data suggests it is a useful and safe agent for the use in the long-term [57, 58]. Results from a pooled analysis, including studies lasting 2 to 24 weeks, showed a difference of 3.6 kg over placebo [59]. There are no long-term studies, however the most relevant studies evaluating phentermine are those with the combination with topiramate, which will be reviewed later in paper.

## Fixed-dose combination of drugs: naltrexone plus bupropion and phentermine plus topiramate

Considering that obesity is a chronic disease and has a complex pathophysiology, targeting multiple pathways at once could lead to better results than targeting just one component. Combination therapy is widely accepted in other chronic metabolic diseases, such as hypertension and diabetes, but much less in obesity. Among the advantages, we can cite an increased therapeutic efficacy due to the synergistic or additive action of the combined drugs, blockade of compensatory mechanisms that lead to a weight loss plateau, and the possibility of using lower doses to minimize the chances of adverse effects. However, there are also disadvantages, including undesirable drug interactions, more contraindications, higher costs and, specifically in combination treatments, dosage inflexibility [60]. The potential for combination therapies was supported by a study that demonstrated a higher probability for a fixed combination to advance from phase I to approval than a drug studied in monotherapy [61]. To date, two fixed-dose combinations have been studied and approved in some countries.

### Naltrexone plus bupropion

The combination of naltrexone with bupropion (NB) has been available for chronic management of obesity in the US and Europe since 2014 and has recently been approved in Brazil. Naltrexone is an antagonist of opiate receptors, and bupropion is a dopamine and noradrenaline reuptake inhibitor. The mechanism underlying the reduction in appetite from bupropion is not entirely understood; it may decrease food reward or directly affect the hypothalamus, increasing satiety [62]. Naltrexone alone does not provide clinically significant weight loss; however, the association with bupropion shows a synergistic action on rewarding pathways, enhancing the reduction of food intake and increasing weight loss when compared to bupropion alone [63–65].

Patients with obesity receiving NB (32 mg/360 mg) combined with a hypocaloric diet (500 kcal deficit per day) and increased physical activity for 56 weeks lost 8.1% of their body weight compared with 1.8% for the placebo group [66]. Similar results were reported by a double-blind RCT, including 1,496 patients with obesity and controlled hypertension and/or dyslipidemia [67]. Patients who underwent NB treatment (32 mg/360 mg) combined with a hypocaloric diet (500 kcal deficit per day) and increased physical activity for 56 weeks had a mean body-weight loss of 8.2% versus 1.4% with placebo (p < 0.001). NB as an adjunct treatment of intensive behavior modification yielded a 9.3 ± 0.4% body-weight loss compared with 5.1 ± 0.6% for placebo (p < 0.001) [68].

Patients with obesity and T2DM were also shown to achieve weight loss when treated with NB. In a double-blind RCT (n = 505), NB combined with a hypocaloric diet (500 kcal deficit per day) and an increase in physical activity for 56 weeks led to a mean change in body weight of -5.0% versus -1.8% with placebo (p < 0.001) [69].

None of the trials cited above showed data on participants with a weight loss greater than 20%. Data regarding changes in body composition are limited.

The results from a meta-analysis including four RCTs with NB treatment showed that the OR for losing at least 5% of body weight was 3.96, and 4.19 for losing at least 10% of body weight [42]. The placebo-subtracted weight loss was 4.9 kg after one year of treatment [42].

The most frequent adverse events reported in the RCTs with NB treatment were nausea, headache, and constipation [66, 67]. Cardiovascular safety still needs to be investigated by a RCT, but a recent systematic review and meta-analysis found no association between the use of naltrexone, bupropion, or their combination and the incidence of major cardiovascular adverse events compared with placebo [70]. Nevertheless, patients with hypertension or other cardiovascular diseases should be aware of the small but not trivial sympathomimetic effects of bupropion, as it can increase blood pressure and heart rate [65]. Bupropion is contraindicated in patients with a history of seizures, as it lowers the seizure threshold [65]. Nausea and vomiting are the most common adverse effects of naltrexone; it should be used with caution in patients with a history of opioid use, as naltrexone can reduce the effects of opioids and even lead to withdrawal syndrome [65].

### Phentermine plus topiramate

Phentermine combined with topiramate (PHEN/TPM) has been approved in the US since 2012, but its use is not approved in Brazil. Phentermine is an alpha-adrenergic receptor antagonist showing anorexigenic properties [71]. As an amphetamine analog, it stimulates the release of norepinephrine acting on the hypothalamus, increasing leptin [71, 72]. Topiramate is an anticonvulsant agent that acts on gamma-aminobutyric acid (GABA) receptors. The mechanisms involved in weight loss are not clear [72], although it is known to reduce food intake (especially cravings) and compulsive eating [65]. The idea of combining both drugs is to induce synergistic effects and reduce the overall side effects, as they have some opposing side effects [65].

The CONQUER trial was a double-blind phase-3 RCT (n = 2,487) evaluating PHEN/TPM 15/92 mg combined with diet and physical activity for treating patients with obesity and more than two weight-related comorbidities [73]. This study showed the superiority of PHEN/TPM with a reduction of 8.6% in body weight compared with -1.2% with placebo (p < 0.0001) [73].

The 52-week extension study (the SEQUEL study; n = 676) evaluated maintenance treatment with PHEN/TPM 15/92 mg [74]. At the end of 108 weeks, patients treated with PHEN/TPM lost a mean of 10.5% of their initial body weight while those treated with a placebo lost 1.8% (p < 0.0001).

In a double-blind parallel-group RCT (n = 1,267), patients with class II and III obesity (BMI ≥ 35 kg/m^2^) treated with PHEN/TPM 15/92 mg combined with a daily 500-kcal deficit diet, increased water consumption, and increased physical activity for 56 weeks lost 10.9% of their initial body weight compared with 1.6% for the placebo group (p < 0.0001) [75].

A meta-analysis concluded that the OR for achieving at least a 5% body-weight loss with PHEN/TPM was 9.22, and 11.40 for achieving at least a 10% body-weight loss compared with a placebo [76]. After one year of treatment, the placebo-subtracted weight loss was 8.8 kg [42]. None of the trials cited above reported the evaluation of lean- and fat-mass reduction.

According to data from a meta-analysis, PHEN/TPM is associated with dysgeusia, paresthesia, hypoesthesia, constipation, dry mouth, reduced attention, irritability, and dizziness [77]. Topiramate should be used with caution in women of childbearing age, as it can lower contraceptive efficacy, and it is associated with a 1.5% increase in cleft palate malformations [65]. There is currently no available information on the cardiovascular safety of PHEN/TPM. However, since phentermine has a sympathomimetic action and is associated with an increase in heart rate, it should not be used in individuals with established cardiovascular disease or a history of arrhythmias.

Before semaglutide, PHEN/TPM was the AOM shown to have higher weight-loss efficacy in RCTs and the only AOM that reached the mean 10% threshold. However, it was shown to have a relatively low prescription rate in the US [22].

## Central action with activity on Nutrient stimulated based hormones (NUSH) [78]: liraglutide and semaglutide

The success of glucagon-like peptide 1 receptor agonists (GLP-1RAs) in the treatment of obesity and T2DM, including a direct impact on cardiovascular and renal outcomes in T2DM, led to an increase in potential drugs with higher efficacy, including pure GLP-1RAs or dual or triple agonists combined with other gastrointestinal peptides [79].

### Liraglutide

Glucagon-like peptide 1 (GLP-1) is an intestinal peptide hormone that participates in satiety mechanisms, in addition to its incretin action that improves blood glucose control. GLP-1 decreases appetite and food intake by stimulating satiety signaling in the brain, directly affecting the hypothalamus, vagal nerve stimulation, and other afferent neural pathways [80]. Liraglutide, in addition to its T2DM indication, is widely approved for obesity treatment in adults and adolescents 12 years old and older, whose body weight is greater than 60 kg and who have an adjusted BMI ≥ 30 kg/m^2^.

A RCT including adolescents with obesity demonstrated a significantly greater reduction in BMI with liraglutide compared with placebo; 43.3% vs. 18.7% had at least a 5% reduction in BMI, and 26.1% vs. 8.1% had at least a 10% reduction [81].

The SCALE (Satiety and Clinical Adiposity—Liraglutide Evidence) programme comprise four phase 3a clinical trials that evaluated the use of liraglutide and weight loss in individuals with and without diabetes [82–86]. A double-blind RCT evaluated liraglutide 3.0 mg in the treatment of obesity for 20 weeks (n = 564), with a two-year extension open-label period (n = 268) [82]. Liraglutide showed superior weight loss over orlistat and placebo, and decreased body fat by 15.4% and lean mass by 2% after 20 weeks of treatment [82]. Moreover, it resulted in sustained weight loss, as > 85% of the patients with a > 5% body-weight loss in the first year maintained that loss in the second year. The mean weight loss with liraglutide was 10.3 ± 7.1 kg over the two-year follow-up. Another RCT (n = 3,731) with 56 weeks of treatment showed similar results, being liraglutide superior to placebo in 5% and 10% weight loss [83]. In patients with obesity and T2DM (n = 846), the use of liraglutide 3.0 mg for 56 weeks combined with a daily 500-kcal-deficit diet and increased physical activity yielded a mean weight loss of 6.0% versus 2.0% for those using a placebo [84]. To evaluate the efficacy of liraglutide after weight loss, 422 patients randomly received either liraglutide 3.0 mg or placebo for 54 weeks after losing ≥ 5% body weight with a low-calorie diet (1200–1400 kcal/day) and regular physical exercise [85]. The additional mean weight loss with liraglutide was 6.2% versus 0.2% with placebo (p < 0.0001). None of the trials reported weight loss greater than 20%.

Liraglutide was also studied in post-bariatric patients with weight regain (n = 117) [86]. The mean weight loss was 5.5% of total body weight after 7.6 months using liraglutide 3.0 mg combined with dietary changes and exercise. Recently, liraglutide was studied alone or in conjunction with physical activity to maintain weight loss after an 8-week very low-calorie diet in which the mean achieved weight loss was 13.1 kg [87]. After one year of treatment, patients treated with liraglutide alone or with exercise continued to lose weight, while those with exercise alone or placebo gained it.

A meta-analysis including two RCTs with liraglutide showed that the OR were 5.54 and 4.99 for achieving a ≥ 5% and 10% body-weight loss, respectively; the weight loss (placebo-subtracted) after one year of treatment was 5.27 kg [42]. Another meta-analysis with 31 studies and 8,060 participants showed a 4.19 kg mean difference in weight loss between the liraglutide group and the placebo group [88].

The liraglutide trial with the longest duration lasted three years (n = 2,254) and showed a greater mean weight loss for the liraglutide group (-6.1%) than for the placebo group (−1.9%) and sustained weight loss during the three years [89].

The most frequently reported adverse events related to liraglutide were nausea and vomiting [82]. The effects of Liraglutide 1.8 mg were studied in the LEADER cardiovascular outcome trial, and the results showed lower cardiovascular events in the liraglutide arm, confirming noninferiority as well as a superiority benefit, with reduction of cardiovascular death, nonfatal stroke, and myocardial infarction in patients with T2DM (HR 0.87; 95% CI 0.78, 0.97; p < 0.001) [90, 91]. A post hoc analysis from SCALE RCTs (n = 5908) showed that treatment of obesity with liraglutide 3.0 mg does not increase the risk of cardiovascular events (HR 0.42; 95% CI 0.17, 1.08) [84], although the total number of events was too small to draw definitive conclusions. Moreover, a meta-analysis evaluating GLP-1RAs (n = 56,004 patients with T2DM), including liraglutide, demonstrated that this class of drugs is safe, especially regarding cardiovascular outcomes [92]. The results from the pooled analysis including different GLP-1RA receptor agonists showed a 12% reduction in the risk of major cardiovascular events (MACEs) (HR 0.88; 95% CI 0.82, 0.94; p < 0.0001) and a reduction in all-cause mortality (HR 0.88; 95% CI 0.83, 0.95; p = 0.001) and did not increase the risk of acute pancreatitis or pancreatic cancer [92].

### Semaglutide

Semaglutide is a GLP-1RA that has been shown to have the most significant effect on body weight in the treatment of T2DM, thus inspiring studies on obesity in patients without diabetes. Studies using higher doses of semaglutide in this population achieved the mean 15% weight-loss threshold for the first time, thereby changing the landscape of obesity treatment and bridging the gap between patient expectations and results.

Semaglutide is approved in the treatment of T2DM, and the US, Europe and Brazil recently approved an injectable 2.4 mg presentation to treat obesity in adults. As a GLP1-RA, semaglutide directly affects areas of the hypothalamus and the hindbrain related to enhancing satiety and reducing rewarding signaling [93]. Semaglutide suppressed food intake in treated mice with obesity, yielding weight loss of up to 22% along with fat-mass reduction [93].

Semaglutide bridges the gap between the desired and the achieved weight loss, as a substantial number of patients achieved a significant loss of ≥ 20% of their bodyweight. In a phase-2 trial (n = 957), semaglutide was compared with liraglutide 3.0 mg and placebo combined with a daily 500 kcal deficit diet and physical exercise for 52 weeks [94]. Patients received different daily doses of semaglutide (0.05 mg, 0.1 mg, 0.2 mg, 0.3 mg, or 0.4 mg/day), equivalent to 0.25, 0.50, 1.0, 1.7, and 2.4 mg/week according to pharmacokinetic calculations [94, 95]. Patients treated with 0.4 mg/day semaglutide, corresponding to 2.4 mg/week, had a 13.8% body-weight loss at the end of the study. Patients treated with liraglutide lost 7.8% and those treated with a placebo lost 2.3%. Semaglutide showed a superior percentage of weight loss at all dosages compared with liraglutide and placebo. Moreover, a significant percentage of patients (37%) lost ≥ 20% of body weight.

In the STEP program, semaglutide was widely studied in various scenarios in the population with obesity. The program included several phase-3 trials evaluating the efficacy of subcutaneous weekly injections of semaglutide 2.4 mg for treating patients with obesity [95–100].

Common elements of the STEP 1–4 trials were the duration of 68 weeks and the 16-week-long regimen of dose escalation [95–98]. The treatment was initiated with 0.25 mg semaglutide and was escalated every 4 weeks to the subsequent dosing levels of 0.5 mg, 1.0 mg and 1.7 mg until reaching the target dose of 2.4 mg. Safety and tolerability were assessed up to week 75 (68 weeks on-treatment and 7 weeks off-treatment follow-up period).

**Fig. 2:**
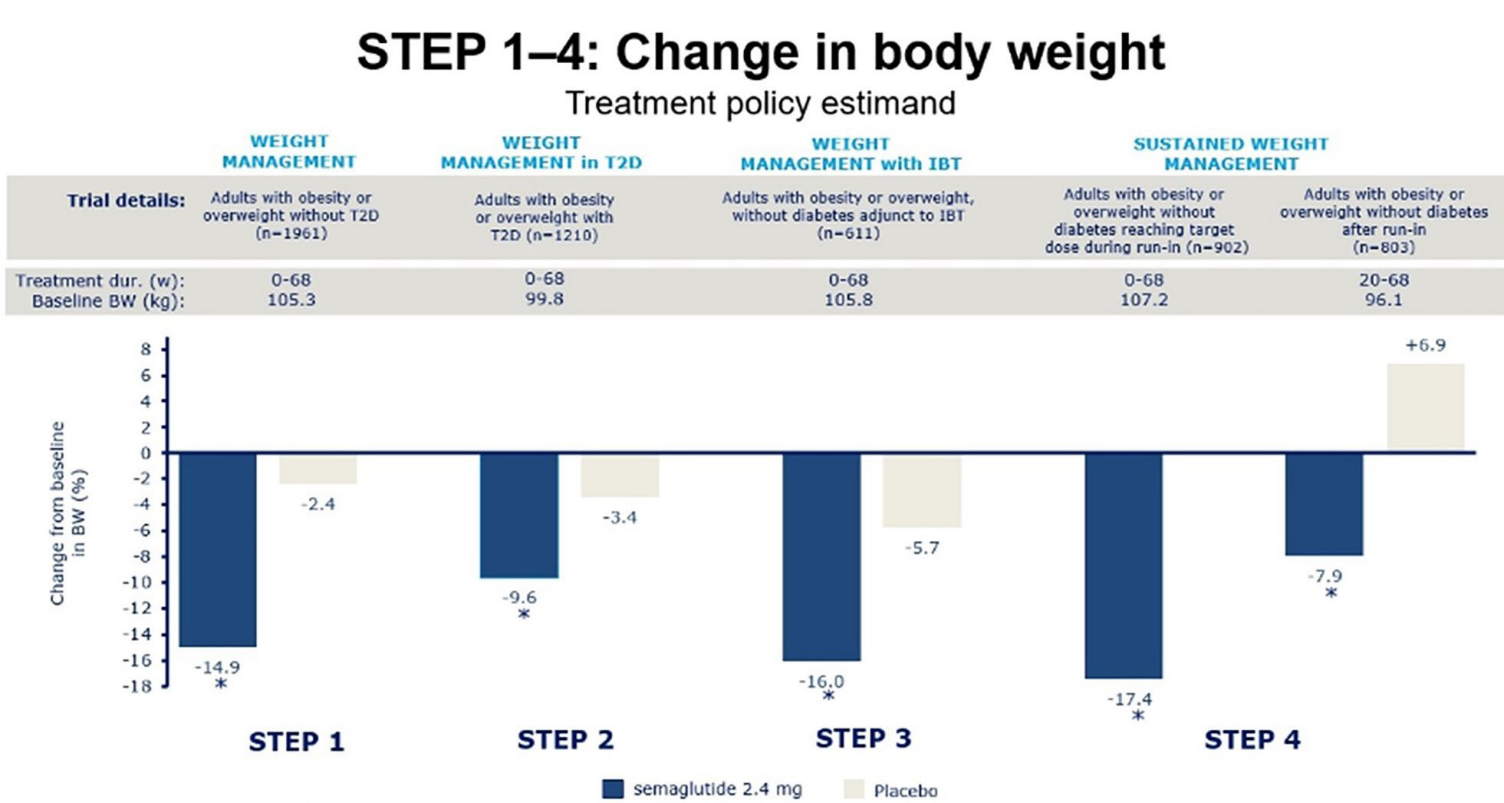
Change in body weight with semaglutide 2.4 mg: STEP trials [95–98]

STEP 1 was a phase-3a randomized, double-blind, multicenter, placebo-controlled trial in 1,961 adults with obesity [95]. The main objective of STEP 1 was to evaluate the weight-management effects of semaglutide 2.4 mg versus a placebo combined with lifestyle interventions such as counseling, 500 kcal reduced daily energy intake and increased energy expenditure (150 min/week physical activity) [95]. Factors such as a ≥ 10% or ≥ 15% reduction in body weight, changes in waist circumference, systolic blood pressure, and physical functioning scores were also evaluated as secondary endpoints. In that study, patients (n = 1,306) with obesity (BMI ≥ 30 kg/m^2^) or who were overweight (BMI ≥ 27 kg/m^2^, with one or more weight-related comorbidities) received either semaglutide 2.4 mg or placebo, in addition to lifestyle changes, for 68 weeks. A total of 94.3% of the participants completed the trial. At the end of the trial, the mean percentage of body weight lost was 14.9% with semaglutide versus 2.4% with placebo (p < 0.001). The mean change in body weight (kg) is illustrated in Fig. 2. Investigators evaluated the body composition changes of a subgroup of the STEP 1 trial [95]. The overall weight loss in the subgroup was 17.4 kg in the semaglutide group, with 10.4 kg of fat mass (60%) and 6.92 kg of fat-free mass (40%); there was an increase in the proportion of lean mass (+ 3.04% in absolute numbers). More studies are warranted to evaluate whether specific lifestyle changes could reduce the loss of lean mass with semaglutide. The secondary endpoints of the study, cardiometabolic risk factors such as waist circumference, blood pressure, glycated hemoglobin levels, and lipid levels, baseline C-reactive protein, and the proportion of participants with normoglycemia, were significantly improved in the semaglutide group compared with the placebo group. The magnitude of these improvements is presumed to be proportional to weight loss. Regarding safety, both groups reported a similar number of adverse events (89.7% in the semaglutide group and 86.4% in the placebo group). Gastrointestinal disorders were the most common and were more frequent in the semaglutide group (74.2% vs. 47.9%). Serious events (also mainly gastrointestinal disorders) were reported in 9.8% and 6.4% of the cases and led to discontinuation of the trial in 7.0% of the cases in the semaglutide group vs. 3.1% in the placebo group.

In the follow-up of the STEP 1 trial, which included 327 participants with a mean weight loss of 17.3% in the semaglutide group and 2.0% in the placebo group, after the discontinuation of the treatment and lifestyle interventions, participants regained two-thirds (11.6 vs. 1.9 percentage points) of their previous lost weight, resulting in net losses of 5.6% vs. 0.1% by week 120 [101]. These data confirm that obesity is a chronic and recurrent disease, and medications will only be useful when used continuously; this concept is not different from that of hypertension, diabetes, or dyslipidemia.

The double-blind, double-dummy, placebo-controlled STEP 2 study evaluated patients with BMI ≥ 27 kg/m^2^ and T2DM (n = 1210) [96]. This was the only study included in the STEP program that involved patients with diabetes and, as such, the first trial to show clinically relevant weight loss related to a once-a-week subcutaneous injection of semaglutide 2.4 mg in people with overweight or obesity and T2DM. Patients treated with weekly subcutaneous injection of semaglutide 2.4 mg for 68 weeks showed a – 9.6% reduction in their initial body weight versus a – 7.0% reduction with 1.0 mg of semaglutide and a – 3.4% reduction with a placebo (p < 0.0001). A greater percentage of patients (13.3%) also achieved a ≥ 20% body-weight loss with semaglutide 2.4 mg, versus 4.7% in the semaglutide 1.0 mg group and 1.6% in the placebo group. The mean change in body weight (kg) is illustrated in Fig. 2.

In the STEP 3 trial, patients who were overweight (BMI ≥ 27 kg/m^2^) with one weight-related comorbidity or with obesity (BMI ≥ 30 kg/m^2^), without diabetes or an HbA1c ≥ 6.5%, received semaglutide 2.4 mg or placebo once a week combined with intensive behavioral therapy (IBT), characterized by an initial low-calorie diet (1000 to 1200 kcal/day), followed by a hypocaloric diet (1200 to 1800 kcal/day), 200 min/week of moderate-intensity physical activities, for 68 weeks [97]. IBT was included as an adjunct to treatment in STEP 3, as previous trials have demonstrated an increase in weight loss when combined with medications approved for chronic weight management compared with IBT alone [102, 103]. Patients treated with semaglutide had a 16% mean body-weight loss versus 5.7% with placebo (p < 0.001) [97]. The percentage of patients to lose ≥ 20% of body weight was 35.7% in the semaglutide group versus 3.7% in the placebo group (p < 0.001). The mean change in body weight (kg) is illustrated in Fig. 2. The results obtained by the semaglutide group, but not by the placebo group, were similar to the findings from the STEP 1 trial [95], where the participants did not undergo an intensive lifestyle modification program. The similarity of the weight loss achieved by the semaglutide group participants in both trials, despite the different lifestyle therapies used, allows us to speculate that such intensive changes in lifestyle implemented in IBT may not increase the effectiveness of AOMs with semaglutide profiles. For patients using semaglutide 2.4 mg, a nonintensive lifestyle modification program may be sufficient.

STEP 4 was a phase-3a randomized, double-blind, multicenter, placebo-controlled trial with 902 adults with obesity (BMI ≥ 30 kg/m^2^) or overweight (BMI ≥ 27 kg/m^2^) with at least one weight-related comorbidity and without diabetes or an HbA1c ≥ 6.5% [98]. The study aimed to evaluate how continued treatment with semaglutide 2.4 mg compared to withdrawing the treatment affects weight maintenance in people with overweight or obesity after significant weight loss. After 20 weeks and a mean bodyweight loss of 10.6% with semaglutide, individuals who continued with semaglutide achieved an additional loss of 7.9% of body weight by week 68 versus 6.9% weight gain with placebo (p < 0.001). Greater weight loss with continued treatment was associated with early response at week 20; however, nonearly responders could also achieve a ≥ 5% weight loss by week 68 if semaglutide treatment was continued. The mean change in body weight (kg) is illustrated in Fig. 2. These results are consistent with the chronic and relapsing nature of obesity and indicate that continued treatment with semaglutide 2.4 mg is required for sustained weight loss.

The STEP 5 trial was the longest of all the trials from the STEP program evaluating long-term weight management with semaglutide 2.4 mg versus placebo and comparing the effects on the control of eating [99]. With a two-year duration, STEP 5 included 304 patients with obesity (BMI ≥ 30 kg/m^2^) or overweight (BMI ≥ 27 kg/m^2^) with at least one weight-related comorbidity except T2DM. At the end of the study, patients in the semaglutide group had lost 15.2% of their body weight versus -2.6% in the placebo group, resulting in an estimated treatment difference of – 12.6% points (95% CI – 15.3, – 9.8; p < 0.0001). In the semaglutide group, 36.1% lost ≥ 20% of body weight versus 2.3% in placebo group. The semaglutide group presented superior short- and long-term control of eating, which resulted in significant weight loss associated with clinically and statistically significant decreases in blood pressure, metabolic parameters and inflammation, as measured by ultrasensitive C-reactive protein. A total of 79.7% of the participants from the semaglutide group who began the trial with prediabetes reached normoglycemic values by the 104th week. This number was significantly lower (37.0%) in the placebo group. These data support that the substantial weight loss reported during 68 weeks with semaglutide 2.4 mg can be maintained when continued up to at least 104 weeks, proving the durability of the effects on energy intake, supporting its long-term effectiveness for the treatment of obesity, and reassuring its safety profile. The mean weight loss of ~ 15% achieved with semaglutide 2.4 mg at week 104 in STEP 5 exceeds the weight loss reported at similar time points in trials with other pharmacotherapies for weight management in adults with overweight or obesity.

The STEP 8 trial, including 338 patients with obesity (BMI ≥ 30 kg/m^2^) or overweight (BMI ≥ 27 kg/m^2^) with at least one weight-related comorbidity, is one of the few head-to-head studies in the field of obesity research, comparing semaglutide 2.4 mg/week with liraglutide 3.0 mg/day and placebo combined with counseling, a daily 500-kcal-deficit diet and 150 min/week physical exercise for 68 weeks [100]. Patients treated with semaglutide showed a 15.8% body-weight reduction versus 6.4% for the liraglutide group and 1.9% for the placebo group, with an estimated treatment difference of -9.4% between semaglutide and liraglutide (95% CI −12.0, −6.8; p < 0.001). 38.5% of patients treated with semaglutide lost ≥ 20%, versus 6.0% with liraglutide (p < 0.001).

Semaglutide has also been studied in an adolescent population, an age group in which the obesity prevalence has been rising in an even higher proportion than in adults, and in which weight stigma, with all its health consequences, can be very prevalent and damaging. The STEP Teens study was a double-blind, parallel-group, randomized, placebo-controlled trial in adolescents (aged 12–18 years) with obesity (BMI in the 95^th^ percentile or higher) or with overweight (a BMI in the 85^th^ percentile or higher) and at least one weight-related coexisting condition [104]. The main objective was to evaluate semaglutide 2.4 mg versus placebo combined with lifestyle interventions. Factors such as a ≥ 10% or ≥ 15% reduction in body weight, change in waist circumference, systolic blood pressure, and physical functioning scores were also evaluated as secondary endpoints. After 68 weeks, the semaglutide group showed a significantly higher proportion of participants (73%) who had weight loss of 5% or more compared with the placebo group (18%) (estimated OR, 14.0; 95% CI 6.3, 31.0; p < 0.001). Those results appear similar or even superior to what has been achieved in adults. Moreover, a post hoc analysis of the STEP Teens study demonstrated that > 40% of the participants with semaglutide treatment achieved an improvement of ≥ 2 BMI categories, compared to 3.4% in the placebo group [105]. Almost three-quarters (73.7%) of the participants treated with semaglutide improved ≥ 1 BMI category (19.0% in the placebo group), and almost half of the participants (44.9%) no longer had obesity at week 68, which can potentially change the natural history of obesity and its complications.

The STEP program demonstrates the impact of semaglutide on weight loss in adults and adolescents with obesity or overweight. Its effects include a decrease in hunger, resulting in lower energy intake (−47.1%) compared with placebo (18.6%), a decrease in food consumption, and an increase in satiety (p < 0.02) [106]. Patients treated with semaglutide have higher postprandial appetite suppression, less food craving, and more control of eating (p < 0.05) [106]. Semaglutide also reduces the desire for sweet, savory, and dairy foods (p < 0.05) [99, 106]. This effect persists in long-term treatment, as the results from the longest trial evaluating treatment with semaglutide for two years (STEP 5 trial) showed that control of eating was significantly better with semaglutide than with placebo, with better craving control (p = 0.0082) and less craving for savory foods (p = 0.0010) [99].

Gastrointestinal-related side effects, especially nausea, were the most commonly reported adverse events in the RCTs, usually with mild-to-moderate intensity [95–101]. Data are available for the cardiovascular safety of injectable semaglutide with 0.5 mg to 1.0 mg/week doses in patients with T2DM [90]. The use of semaglutide in this population with established cardiovascular disease led to a significantly 26% lower rate of the primary outcome; however, those data are only hypothesis-generating because the trial was not sufficiently powered to detect significant differences in cardiovascular outcomes.

One randomized, double-blind, parallel-group trial—the Semaglutide Effects on Cardiovascular Outcomes in People With Overweight or Obesity (SELECT) study –evaluated the risk of MACEs with a 5-year follow-up [107]. It included 17,604 adults overweight or obese with an average age of 61.6 years and BMI of 33.34 kg/m^2^ without diabetes but with established cardiovascular disease. The results showed a 20% reduction in MACEs (6.5% versus 8.0% with placebo, HR 0.80, 95% CI 0.72–0.90, p < 0.001), a result with potential to change dramatically the obesity treatment scenario in the long-term.

Another phase-3 trial evaluated the effect on weight loss and heart failure-specific symptoms of semaglutide 2.4 mg versus placebo in 529 adults with obesity and heart failure with preserved ejection fraction [108]. Patients treated with semaglutide had fewer symptoms and physical limitations and greater weight loss (−13.3%) compared with those treated with placebo (−2.6%) and there is evidence that the effect are largely mediated by weight loss [109].

An ongoing phase-3 trial will evaluate the effect on weight loss and heart failure-specific symptoms, health status and health-related quality of life of semaglutide 2.4 mg versus placebo, both with standard of care, in 610 adults with obesity and T2DM [110].

Other ongoing trials from the STEP program are one investigating the effect of semaglutide 2.4 mg versus placebo on body weight and knee osteoarthritis-related pain [111] and semaglutide 2.4 mg versus placebo on reversal of prediabetes [112].

## Other potential drugs for weight management

Several other drugs are being studied for obesity [113, 114], including other GLP-1RAs and combinations [115–117], but it is not in the scope of this article to discuss promising drugs. We will briefly discuss tirzepatide, recently approved in US (November 2023) but not yet in Brazil, with a robust phase 3 program in obesity.

Tirzepatide is a once-weekly glucose-dependent insulinotropic polypeptide (GIP) and GLP-1 agonist. The reason why GIP potentiates GLP-1 weight loss is still subject to speculation. Two phase-3 clinical trials, SURMOUNT-1 and SURMOUNT-2 showed its superiority over placebo [117–119]. In SURMOUNT-1, 2539 individuals with obesity or overweight with a weight-related comorbidity (non-diabetes) were randomized to receive tirzepatide or placebo, for 72 weeks [117, 118]. At the end of the study, the mean weight loss with the higher dose (15 mg) of tizerpatide was 20.9% versus 3.1% with placebo. The drug was considered safe, as the most common adverse event was gastrointestinal, from mild to moderate intensity. In SURMOUNT-2, 938 participants were individuals with BMI ≥ 27 kg/m^2^ and T2DM receiving tirzepatide or placebo, for 72 weeks [119]. The weight loss at the end of the follow-up was greater with tirzepatide than placebo (-14.7% with 15 mg/week versus 3.2%, respectively). The most common adverse events were nausea, diarrhea, and vomiting, from mild to moderate intensity.

## Higher weight loss: what does this mean for the treatment of obesity? are there new concerns that could arise?

**Fig. 3:**
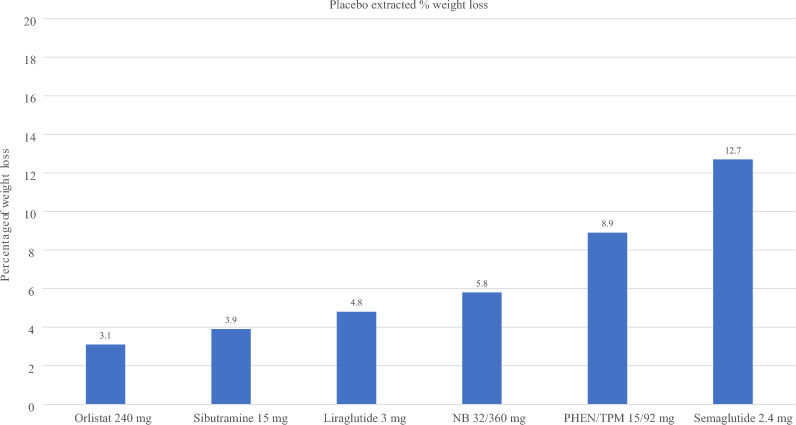
Reported efficacy of anti-obesity agents. *NB* Naltrexone combined with bupropion, *PHEN/TPM* Phentermine combined with topiramate

**Fig. 4:**
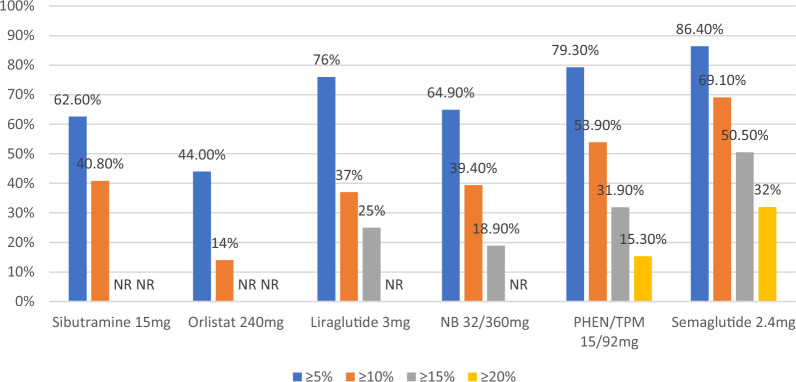
Efficiency of anti-obesity drugs in reducing body weight by 5% to 20%. *NR* not reported, *NB* Naltrexone combined with bupropion, *PHEN/TPM* Phentermine combined with topiramate

Figure 3 illustrates the mean placebo-subtracted weight loss of AOMs from RCTs with at least 24 weeks of use in a noncomparative manner to provide an overview of the studies. Fig. 4 shows the corresponding weight loss for each drug according to the percentage. Since there are only a few head-to-head studies available to compare the efficacy of different drugs, the graphs should be seen as merely illustrative.

The STEP trial results are a landmark in the treatment of obesity, as they reach the 17% weight-loss threshold, and a large proportion of individuals achieved weight loss over 20%, which could be compared to the results of sleeve gastrectomy [120]. This new scenario and the prospect of even more potent drugs in the near future have prompted many researchers to speculate whether new AOMs could replace bariatric surgery. Although the overall results suggest that this could be the case for at least a fraction of individuals with obesity, there is still a widespread stigma against the chronic use of AOMs [20], and most individuals tend to interrupt treatment after reaching a weight plateau or achieving the target weight. Withdrawal from the treatment will lead to weight regain, as seen in the follow-up of the STEP 1 trial [95], whereas the recovery of weight lost after bariatric surgery tends to be much lower than with clinical treatments [121]. Therefore, changing this scenario will only be possible by a radical change in public and medical perspectives, recognizing obesity as a chronic disease that needs chronic treatment. Whether repeated weight loss followed by weight regain is detrimental to health is still a matter of debate, but it could lead to changes in body composition (increase in the proportion of fat to lean mass) that could, in the long term, at least make it more difficult to achieve further reductions in body weight [121, 122]. Other potential negative consequences of major weight loss, such as bone loss and vitamin deficiencies, should also be better scrutinized in future trials [123–126].

Last, the SELECT study is a major turning point in the obesity field [109]. With a drug aiming to treat obesity showing cardiovascular benefit, with an impressive 20% reduction in cardiovascular events, it could considerably change the body of evidence in favor of a wider prescription and significant impact on obesity comorbidities, such as myocardial infarction, ischemic or hemorrhagic stroke, symptomatic peripheral arterial disease, or chronic heart failure. We hope that the reduction in hard outcomes reduce obesity treatment stigma and leads to wider availability of pharmacotherapy by public health systems, provided it is cost-effective.

In summary, having set the scene for several medications in the treatment of obesity, it is important to note that there is no “one size fit all” approach to obesity, and having a higher armamentarium is useful so we can find the best drug for each patient. Each drug has its own characteristics, and even with more powerful drugs, there is a substantial rate of non-responders [95]. There have been suggestions of a “phenotype-based treatment” [127], which demonstrated, at least in one clinical trial, to be useful, but the exact way to phenotype and the exact drugs to be prescribed in each scenario is still a question of debate, and guidelines suggest different pharmacological approaches depending on clinical characteristics [128]. It is possible to use drugs with higher efficacy as first-line treatment and reserve older, less potent, medications for those non-responders, as well as consider combination therapies [60]. Moreover, with hard outcomes data, and cardiovascular benefit, give priority to semaglutide in patients with established cardiovascular disease is warranted, as well. It is not the scope of the article to fully discuss in which profile each drug will be useful, but highlight that, even with better new drugs, older options would still be useful in specific scenarios.

## Conclusions

Although obesity is a chronic disease associated with several health consequences and reduced longevity, it is rarely treated. There are several reasons why AOMs are underused and stigmatized. In addition to the stigma of the disease itself, AOMs represented a relatively modest mean weight loss, much below the patients’ and physicians’ expectations, which generally leads to early discontinuation of treatment. At the same time, several drugs were banned in the past, as AOMs have been linked to increased health risks. This situation is possibly changing with the discovery of new molecules, such as semaglutide and tirzepatide, resulting in greater weight loss and filling the gap between reality and expectations, with mean weight losses over 15% and a large proportion of patients achieving weight losses over 20% (similar to some bariatric procedures); these drugs have satisfactory overall safety based on data from multiple GLP-1RAs. However, without a change in paradigm regarding how obesity is perceived, AOMs, even if newer and more potent drugs are found, will not be able to achieve their potential. As soon as obesity is treated as a chronic disease requiring continuous clinical treatment, AOMSs can play a significant and widespread role in better control over the disease and improving general health in patients with obesity, whereas bariatric surgery will still be relevant for more severe cases or those who do not respond to clinical treatment.

## Data Availability

Not applicable.

## Abbreviations

AOMs: Anti-obesity drugs
BMI: Body mass index
CI: Confidence interval
HR: Hazard ratio
GABA: Gamma-aminobutyric acid
GDP: Gross domestic product
GIP: Glucose-dependent insulinotropic polypeptide
GLP-1: Glucagon-like peptide 1
GLP-1RA: Glucagon-like peptide-1 receptor agonist
MACEs: Major cardiovascular events
MWAL: Maximal weight achieved in life
NB: Naltrexone combined with bupropion
NUSH: Nutrient stimulated based hormones
OR: Odds ratio
PHEN/TPM: Phentermine combined with topiramate
RCTs: Randomized controlled trials
T2DM: Type-2 diabetes
US: United States
